# Freedom, choice, and the sense of agency

**DOI:** 10.3389/fnhum.2013.00514

**Published:** 2013-08-29

**Authors:** Zeynep Barlas, Sukhvinder S. Obhi

**Affiliations:** Centre for Cognitive Neuroscience and Department of Psychology, Wilfrid Laurier UniversityWaterloo, ON, Canada

**Keywords:** freedom, action awareness, sense of agency, action choice, intentional binding, authorship

## Abstract

The sense of agency is an intriguing aspect of human consciousness and is commonly defined as the sense that one is the author of their own actions and their consequences. In the current study, we varied the number of action alternatives (one, three, seven) that participants could select from and determined the effects on intentional binding which is believed to index the low-level sense of agency. Participants made self-paced button presses while viewing a conventional Libet clock and reported the perceived onset time of either the button presses or consequent auditory tones. We found that the binding effect was strongest when participants had the maximum number of alternatives, intermediate when they had medium level of action choice and lowest when they had no choice. We interpret our results in relation to the potential link between agency and the freedom to choose one’s actions.

## Introduction

The sense of agency is one of the most pervasive aspects of human consciousness and is commonly defined as the sense that one is author of their own actions and sensory consequences (Haggard and Tsakiris, 2009). Although a full understanding of how we experience the sense of agency remains elusive, research conducted in the last decade has been fruitful in providing the basis for greater insight into agentic experience and the processes that might produce it. At the conceptual level, two competing views emphasizing predictive and retroactive processes, respectively, are slowly being reconciled into a unified framework within which to the study the sense of agency (see Moore and Obhi, 2012). Despite this progress, numerous questions about the neurocognitive architecture underlying agency and the type and variety of factors that affect agency, remain.

It has previously been suggested that the subjective experience of agency occurs at both first order (pre-reflective) and higher order (reflective) levels of processing (Bayne and Pacherie, 2007; Gallagher, 2007, 2010; Synofzik et al., 2008a,b; Obhi and Hall, 2011a,b). The distinction between different forms of agentic experience leads to the question of whether the sense of agency originates at the lower level of sensorimotor operations or at a higher level involving interpretive mechanisms. In this respect, two major accounts have been proposed to explain the origins of the sense of agency. The *predictive* account underlines the role of intrinsic and sensorimotor cues, whereas the inferential account posits the contribution of extrinsic cues and high level inferences (Wegner and Wheatley, 1999; Frith et al., 2000; Blakemore et al., 2002; Wegner, 2002, 2003; Frith, 2005; Sato and Yasuda, 2005; Gallagher, 2007).

Many experiments investigating the neurocognitive basis of agentic experience have used explicit judgments as dependent measures of the sense of agency. Such explicit measures most commonly require participants to state how much control they felt over action outcomes (e.g., Sato and Yasuda, 2005; Balslev et al., 2007; Linser and Goschke, 2007; Metcalfe and Greene, 2007; Ebert and Wegner, 2010; Wenke et al., 2010) or the actions themselves (e.g., Wegner et al., 2004; Sebanz and Lackner, 2007). In some cases participants are asked to make direct judgments about the cause or source of an effect in contexts where source ambiguity is present (i.e., confederate, computer, or participant themselves could have caused the effect; e.g., Wegner and Wheatley, 1999; Aarts et al., 2005, 2009; Dijksterhuis et al., 2008; Spengler et al., 2009; Obhi and Hall, 2011a,b).

However, applying only such explicit measures is highly prone to contamination by issues such as social desirability, impression management and the limits of introspection on the part of participants (Metcalfe and Greene, 2007; Schüür and Haggard, 2011; Obhi, 2012). Alternatively, other experiments have employed “intentional binding” as a potentially implicit measure of the sense of agency. The intentional binding effect refers to the temporal attraction between the perceived times of actions and effects observed in voluntary actions (e.g., Haggard et al., 2002; Haggard and Clark, 2003; Haggard et al., 2009; Moore et al., 2009; Strother and Obhi, 2009; Strother et al., 2010). Since it was first introduced, intentional binding has sparked great interest, due to its purported link relationship to the sense of agency (see Moore and Haggard, 2010; Moore and Obhi, 2012). Although the quest to fully unveil this relationship requires extensive investigation, the progress made by the recent research has been promising (for a recent review of intentional binding research, Moore and Obhi, 2012).

To move closer to understanding the potential relationship between binding and the sense of agency, one approach is to investigate factors that could feasibly be related to agency and assess whether they affect intentional binding. If such factors do indeed affect binding, it would lend support to the notion that binding and agency are indeed linked in some, albeit complex, way.

Agency and freedom are often considered to be tightly intertwined. That is, agency is thought to be strongest in an “environment of opportunities” (Pettit, 2001). Indeed, if a person cannot freely choose a course of action, the very notion that they are an autonomous agent is undermined. Given this, it might be expected that agency and freedom are related such that increasing levels of freedom to choose a course of action correspond to increasing levels of agency. In their study, for example, Wenke et al. (2010) assessed the feeling of control over action outcomes when the proportion of cued and free trials (25% vs. 75%) and the compatibility between two different subliminal action primes and responses were manipulated. In the cued trials participants were required to perform the cued action where in the free trials they could freely choose one of two actions. The results showed that participants’ feeling of control was greater when the primes were compatible with the action responses, suggesting the effect of facilitating the action selection processes. Of more interest, the control ratings were higher when the proportion of free trials was high (75/25 ratio). This study suggests an intriguing link between one’s freedom to choose an action and their feeling of control over the consequences of their action.

By extension and reducing the general idea of a link between freedom and agency to a testable laboratory task, intentional binding might also be expected to vary with differences in the degree of freedom. Again, agency and freedom are often talked about together and the feeling of freedom has been linked to choice (e.g., Markus and Schwartz, 2010). In this light it is interesting to note that most previous intentional binding experiments have required participants to make a pre-specified action which is followed by a sensory event such as an auditory tone. In such cases, the participant is free to select *when* to make an action, but is not free to select *which* action to make. By simply changing the number of action alternatives that are available to participants, it is possible to parametrically manipulate the “environment of opportunities” (i.e., choice) and thus ascertain the effect that the number of choice alternatives has on intentional binding. The fundamental question is, do more action alternatives produce greater levels of intentional binding than a more constrained choice set, where the agent is less involved in selecting which action to make?

To this end, in the present study we examined how agency as purportedly indexed by intentional binding, is affected when the number of action alternatives is manipulated. To our knowledge, this is the first study that addresses the potential relationship between freedom of action choice and the sense of agency. Accordingly, in the present study participants were requested to make a key press on a seven-button response pad while watching a conventional Libet clock on the screen. They reported their perceived times of key press or the auditory tone that was produced by their key press. In the no choice condition, they were told to press only one specific button on the response pad. In the medium-choice condition, they were free to choose among three buttons and in the high-choice condition they were allowed to press any of the seven buttons. For reports of the timing of actions and effects, we employed a similar paradigm to that of Libet et al. (1983) (see also Haggard et al., 2002; Obhi et al., 2007, 2009).

## Method

### Participants

Twenty-four right handed participants (18 women; age range = 17–22) took part in the study. All participants had normal or corrected-to-normal vision and received partial course credits for their participation. The study was approved by the Research Ethics Board of Wilfrid Laurier University and all participants gave written informed consent prior to beginning the study. One participant’s data was not included in the analyses due to not following the experimental instructions.

### Apparatus and Procedure

The experiment was programmed in Superlab 4.5 (Cedrus Corporation, USA) and ran on a Dell personal computer (3.07 GHz). The stimuli were presented on a 20 inch monitor (1600 × 1200). Participants sat approximately 60 cm away from the computer monitor and the responses were recorded on a laptop by the experimenter. The experiment consisted of baseline and operant conditions in which the number of keys to press (high: 7, medium: 3, no choice: 1) and the critical event (key press, tone) that participants judged the timing were manipulated. Similar to Haggard et al. (2002) study, the baseline condition consisted of single events with either the key presses or the auditory tones. The key press single event condition included seven (high level of choice condition), three (medium level of choice condition) and one (no choice condition) key press choices. In the no choice condition, participants could only press the blue button centrally placed on the response pad. In the medium level of choice condition, they were told to choose one of the three buttons on the right side of the response pad. In the high level of choice condition, participants were free to choose any of the seven buttons on the response pad. When the critical event was the auditory tone, participants did not make any key press but only reported the time when they heard the tone. In the operant conditions, participants’ key press was followed by a 1000 Hz tone (duration: 100 ms, bit rate: 160 Kbps) presented after a delay of 200 ms and they were asked to report the time of either their key press or the tone. The condition (2: baseline, operant) together with the level of action choices (3: High, Medium, No choice) and the critical event (2: Key press, Tone) in total were tested in ten separate blocks with 30 trials each (see Table 1 for a list of different block types). The order of the blocks was randomized across participants. At the beginning of each block, participants were informed which key or keys they were allowed to press and which of the two events’ timing (key press or the tone) they were going to report. Participants completed six practice trials prior to the beginning of each block. Sixty practice trials in total thus were excluded from the data analysis.

**Table 1:** **Mean judgment errors in each condition**.

**Level of Choice**	**Individual Event**	**Mean Judgment Error**	**SD**
**No Choice**	Key press alone	−35.96	67.85
	Key* tone	−12.68	81.19
	Key tone*	−106.12	135.21
**Medium**	Key press alone	−19.24	83.33
	Key* tone	−13.21	63.10
	Key tone*	−141.55	114.60
**High**	Key press alone	−58.19	62.18
	Key* tone	−11.34	83.65
	Key tone*	−137.73	143.22
	Tone alone	−117.44	97.56

For each event and each condition, perceived times were subtracted from the actual time of the corresponding events.

*Indicates which event was reported in terms of its timing in the operant condition.

**Figure 1:**
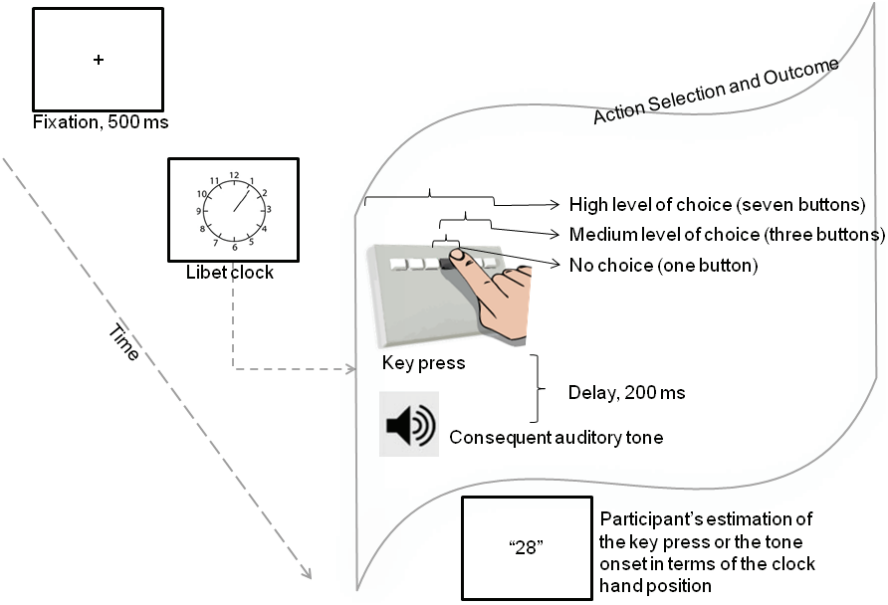
**Trial procedure in the operant condition.** Each trial began with a fixation cross displayed for 500 ms, participants then made a key press at their own pace after the clock started rotating. They were told to press a specific button in the no-choice condition or select one of three (medium level of choice) or seven (high level of choice) buttons on the response pad. The key press was followed by the auditory tone after a delay of 200 ms. In the baseline condition, participants either made a key press without hearing the tone and judged the timing of their key press, or heard the tone which occurred alone and judged the timing of the tone.

Each trial began with a warning signal noting that a new trial will begin, which remained on the screen for 1 s. The fixation cross was then presented for 500 ms and followed by the display of the Libet clock (1.8 cm in diameter) with a minute hand pointing to one of 12 positions marked at 5-minute intervals. Participants were told to report their judgments between 0 (12 O’clock position) and 59, including the intermediate values. The minute hand remained stationary at the center of the screen for 500 ms and then started rotating clockwise at a 2.5 s period. In the baseline—where the single event was the key press only—and in the operant conditions, participants were told to make the key press at their own pace using their right index finger after the clock started rotating. They were instructed not to give stereotyped responses in the high and medium level of choice conditions and not to press the key at predetermined minute hand positions. In the baseline tone-only condition, participants did not make any key press but reported the onset of the tone occurred at a random time (jittered between 200 and 2000 ms) after the clock hand rotation started. The clock continued rotating for about 2000 ms after the participants reported the timing of the critical event. The perceptual times were verbally reported as minute hand positions and recorded by the experimenter on a laptop. At the end of the experiment, participants were debriefed and thanked for their participation in the study (see Figure 1 for a sample trial procedure).

## Results

The experiment comprised a 2 (Condition: Baseline, Operant) × 3 (Level of choice: High, Medium, No choice) × 2 (Critical Event: Action, Tone) repeated measures design. After converting the clock hand judgments to time values in milliseconds, we calculated the judgment errors for each condition as the difference between perceived and actual times of events (Table 1). Trials with key press response time shorter than or equal to 500 ms and with judgment errors three standard deviations away from participant’s average judgment error were excluded from the analysis. In addition, trials in which participants made a key press other than the permitted ones were removed from the data. The exclusion criteria resulted in the removal of 3.06% of all trials (range: 1–11%).

**Figure 2:**
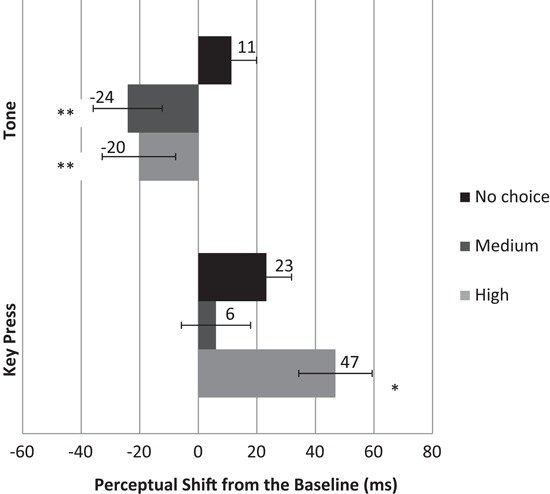
**Mean perceptual shift (difference between the judgment errors in the operant and baseline conditions) for key press (lower) and tone (upper) judgments.** Error bars represent SEM (*indicates that the perceptual shift for key presses in the high level of choice condition was significantly greater than the medium level of choice and no choice conditions, *p* < .05. The difference between medium level of choice and no choice conditions was not significant, *p* > .05. **Indicates that the perceptual shift for the tone judgments in the high level of choice and the medium level of conditions were significantly greater than no choice condition, *p* < .05. The difference between high and medium levels of choice was not significant, *p* > .05).

We then obtained the perceptual shifts in terms of the difference between judgment errors between operant and the corresponding single event baseline conditions for both key press and tone judgments. For example, the perceptual shift for the high level action choice condition was calculated as the difference between the judgment errors in the operant-high-level condition from the baseline-high-level condition. Similarly, the perceptual shifts for the tone judgments were calculated as the difference between the judgment errors in each choice level-tone judgment condition and baseline-tone only condition. The positive shifts in the key press judgments and the negative shifts in the tone judgments relative to the corresponding baseline conditions demonstrate the temporal attraction, i.e., the intentional binding effect, between actions and effects (Figure 2).

We ran a 3 (Level of choice: High, Medium, No choice) × 2 (Critical event: Key press, Tone) repeated measures ANOVA to examine the effect of having different number of action choices on the perceptual shifts. The analysis revealed a significant main effect of key press choice (*F*(2,44) = 3.359, *p* < .05) and a significant main effect of critical event (*F*(1,22) = 5.148, *p* < .05). The interaction between these factors was also significant (*F*(2,44) = 3.389, *p* < .05). We predicted that binding would be least for the no choice condition, strongest for the high level of choice condition and intermediate for the medium level condition. We thus conducted one-tailed Paired Samples *t* tests to examine the 2-way interaction in more detail.

The *t* tests performed on the perceived times of actions revealed that when participants had high number of choices among which keys they could press, their perceptual shift in key press judgments from baseline condition was moved significantly further toward the tone compared to when they had medium level of choices (*t*(22) = 2.287, *p* < .05) and to when they had no choice (*t*(22) = 1.792, *p* < .05). The difference between medium level of choice condition and no choice condition was not significant (*p* > .05).

With respect to the tone judgments, the perceptual shifts moved toward the perceived action onsets for both medium and high levels of choices. The size of the shift was greater for the medium level than the high level and it was in the opposite direction for the no choice condition. We found a significant difference in the perceptual shifts between high level of choice and no choice conditions (*t*(22) = −2.186, *p* < .05) and also between medium level of choice and no choice conditions (*t*(22) = −2.260, *p* < .05). The difference in the perceptual shifts between high and medium level of choices was not significant (*p* > .05).

**Figure 3:**
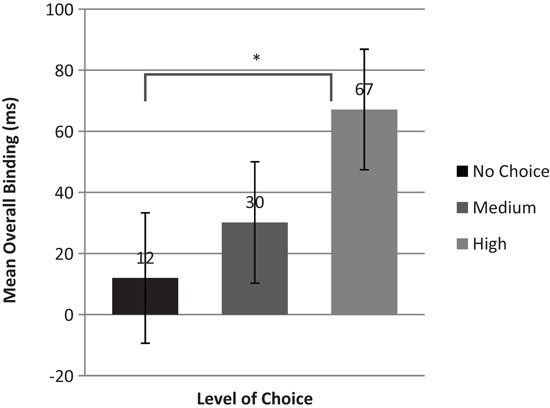
**Mean overall binding as a function of action choice.** Error bars represent SEM (*indicates that binding is significantly greater in the high level of choice condition than in the no choice condition, *p* < .05).

We sought further the effect of choice levels on the mean overall binding by calculating the absolute value of subtraction of the mean key press shift in each condition from the tone shift (Wenke et al., 2009). We conducted a 3 (Level of choice: High, Medium, No choice) repeated measures ANOVA and found a significant main effect of action choice level on overall binding (*F*(2,44) = 3.389, *p* < .05). As expected, we found that overall binding was strongest in the high level of action choice condition, intermediate for the medium level of choice condition and lowest for the no choice condition (Figure 3). We ran one-tailed *t* tests to examine the differences across the three choice levels. The results showed that overall binding in the high level of choice condition was significantly greater compared to no choice (*t*(22) = 1.998, *p* < .05) condition. However, the difference between high level of choice and medium level of choice condition as well as the difference between medium level of choice and no choice conditions were not significant (*p* > .05).

## Discussion

Previous research focusing on different forms of the sense of agency has examined the contribution of various factors including predictive and retrospective processes (see Moore and Obhi, 2012, for a full review of these studies). Action selection is a crucial aspect of the agentic experience and has been shown to enhance the explicit feeling of control when facilitated by the subliminal priming of action alternatives (Wenke et al., 2010). The goal of the present study was to examine how intentional binding would be influenced by different levels of action choice. This is an important question given popular notions about how freedom and agency are intertwined (e.g., Pettit, 2001).

We measured the perceived times of individual key press and tone events separately in both baseline and operant conditions which allowed us to compare the size of the perceptual shift between each level of action choice. First, we found that perceived times of key presses for all levels of choices were shifted forward in time. In the medium level and high level conditions, the direction of the perceived time of the tones was shifted toward the key press whereas, somewhat surprisingly, this was not the case for the no-choice condition. Importantly though, as Figure 2 shows, the overall shift for each individual event (i.e., key press and tone) were in the right direction and demonstrate the intentional binding effect. Of more interest, we found that the degree of overall binding was greatest when participants had the highest level of action alternatives to choose from. In the medium choice condition, binding was not significantly different from the no choice condition, but both these conditions displayed less binding than the high choice condition. Moreover, the magnitude of the binding in three conditions displayed a parametric trend increasing from none to three and seven alternatives (Figure 3). Thus, our results provide support for the notion that a high degree of choice is associated with greater action-effect binding than lower degrees of choice. These results serve to connect the sense of agency to free-choice and are also consistent with the common societal notion that the exercise of personal choice, freedom and agency are intimately intertwined (Hirschmann, 2003; Krause, 2012).

What could be driving our observed effects of choice on intentional binding and by extension, the sense of agency? Given that all possible actions in the set of alternatives produced the same auditory event, our method could be construed as a true test of action selection on the sense of agency. That is, there is no obvious reason why an individual participant may have chosen one action over another, given that the outcome, or reward value of each possible action was fixed. Several explanations are possible.

First, the results we report here are consistent with the finding that intentional binding is stronger when participants specify both the “what” and the “when” component of a pending action, compared to when they specify just one of these dimensions (i.e., “when” or “what”—Brass and Haggard, 2008; Wenke et al., 2009). Participants in the present study were always responsible for specifying the “when” component, but had varying levels of choice about “what” action to make. Specifically, participants were constrained to just one possible action (no choice condition), three possible actions (medium choice condition) or seven possible actions (high choice condition). Thus, in the no choice condition, the action is completely specified externally by the experimenter whereas in both the medium and high choice conditions, the participant must internally specify which action they will ultimately select. By some accounts, the no choice condition can be thought of as more externally triggered than the medium and high choice conditions (see Obhi and Haggard, 2004; Schüür and Haggard, 2011; Obhi, 2012; Schüür and Haggard, 2012). Correspondingly, it has been shown that activation in areas associated with voluntary preparation to act, such as the supplementary motor area (SMA) is greater for actions that are more internally specified than externally specified (Jahanshahi et al., 1995). Thus one broad explanation for our findings is that more internal, endogenous processing prior to action production is linked to higher levels of agency experience, which manifests as greater intentional binding.

Another interesting framework within which to consider our results is based on the affordance competition hypothesis that models behavior as resulting from competition between different representations of potential actions (Cisek, 2007). In this model, action representations are thought of as distributed neural populations that are activated via selective attentional mechanisms (Tipper et al., 1992). By such a view, the action that is finally selected and executed is chosen based on a dynamic reciprocal process operating largely within fronto-parietal circuits which involves mutual inhibition between potential action representations and is subject to biasing by excitatory inputs, some of which arise from cognitive decision making processes (see Cisek, 2007, for a detailed discussion).

Within this framework, we suggest that high, medium and no choice conditions differ in the degree of this dynamic activation and inhibition process that is ultimately responsible for action selection. Specifically, the no-choice condition may not involve the same degree of this dynamic inhibitory and excitatory activity as the high choice condition. We suggest that this difference might result in stronger activation of the representation of the action selected among many, such as in the high choice condition of the present experiment.

This is akin to more endogenous processing being linked to greater agency, as suggested above, with the endogenous activity being specifically the dynamic interplay between excitatory and inhibitory processes during action selection. This explanation also predicts greater binding for the medium choice condition compared to the no choice condition as reported in our study, although the difference was not significant. From the present study, it appears that when seven alternative actions are available, this is sufficient to change the subjective experience of actions compared to when there is no alternative. However three alternatives demonstrate no difference from seven or no alternatives. Clearly, more work is required to determine if this suggestion is tenable, but at the very least, our data do indicate that high choice affects binding in a way that no choice does not.

One might argue that the cognitive load varied across three levels of action choices in our study, which could have contaminated our results. However, as previous studies discussed this concern in detail, (e.g., Haggard et al., 2002) the errors in time judgments in the operant condition are subtracted from their corresponding baseline conditions (e.g., high level of choice action judgment errors in the baseline condition are subtracted from high level of choice action judgment errors in the operant condition) to calculate the perceptual shifts for each event and condition. Since the potential effect of either cognitive or attentional requirements varying across different levels of choice should be present in both baseline and operant conditions, this effect would diminish as a result of the subtraction we used to obtain the perceptual shifts. We thus feel confident in ruling out the effect of differential cognitive load across conditions.

Having demonstrated that a high degree of choice is linked to increased binding, it is important to consider that there are limitations to the present study. For example, we did not assess the explicit sense of agency in this study and so cannot speak to how the number of action choice alternatives might affect the explicit feeling of agency. In addition, we did not manipulate the outcome of the different action alternatives. This is an obvious extension of the current work and would allow for determining the influence of reward on intentional binding and the sense of agency.

Despite these limitations, showing that intentional binding is influenced by the degree of action choice is an important finding and we believe the current study provides a new set of questions relating to how choice affects the sense of agency, which could apply to many domains that extend beyond a fundamental consideration of how the sense of agency arises.

Finally, the current results, along with other recent results from our and other labs, bolster the notion that intentional binding is linked, in some complex way to agentic experience. Specifically, we have previously shown that priming low power reduces binding and activating memories of depression reduces binding, whereas others have shown that less versus more control of an aircraft, when control is shared with an automatic pilot, reduces binding (Berberian et al., 2012; Obhi et al., 2012a,b). Given that these scenarios are all accompanied by real changes in the degree of control that an individual either perceives themselves as having, or actually has, the idea that binding and agency are linked is strengthened. The key is for future work to understand *why and precisely how* the sense of agency and binding are affected by these kinds of manipulations. For now though, the current results reinforce the suggestion that increased personal choice increases agency which could form the foundation for a sense of freedom.

## Conflict of interest statement

The authors declare that the research was conducted in the absence of any commercial or financial relationships that could be construed as a potential conflict of interest.
